# Development of a clinical risk score system for peritoneal dialysis-associated peritonitis treatment failure

**DOI:** 10.1186/s12882-023-03284-1

**Published:** 2023-08-07

**Authors:** Yuhe Mao, Dan Xiao, Shengjing Deng, Shaoqing Xue

**Affiliations:** Department of Nephrology, Meizhou People’ s Hospital, No. 63 Huangtang Road, 514000 Meizhou, Guangdong China

**Keywords:** Peritoneal dialysis-associated peritonitis, Treatment failure, Risk factors, Risk score system

## Abstract

**Objective:**

This study aimed to construct a clinical risk score system for peritoneal dialysis-associated peritonitis (PDAP) treatment failure to provide a theoretical basis for clinical workers.

**Methods:**

A total of 161 PDAP individuals admitted to our hospital were included, among whom 70 cases were in the treatment-improved group and 87 cases were in the treatment failure group. We compared the general condition, clinical manifestations, and laboratory examination indicators of the two groups of individuals, used multivariate logistic regression analysis to identify the factors influencing PDAP treatment failure, and developed a clinical risk score system. The diagnostic performance of the risk score system was evaluated utilizing the receiver operating characteristic (ROC) curve.

**Results:**

Significant differences (*P* < 0.05) were observed between the two groups in terms of contamination, peritoneal fluid culture results, blood urea nitrogen (BUN) level, C-reactive protein (CRP) level, B-type natriuretic peptide (BNP) level, average residual urine (RU) volume, and urea clearance rate (UCR). Multivariate logistic regression analysis showed that BUN level, CRP level, BNP level, average RU volume, and UCR were independent risk factors affecting PDAP patient treatment outcomes (*P* < 0.05). The ROC curve analysis of the risk score system for predicting treatment failure in PDAP individuals showed an area under the curve of 0.895 [95% confidence interval (0.847–0.943)]. The optimal cut-off point was 2.5 points, with corresponding sensitivity and specificity of 88.5% and 74.3%, separately.

**Conclusion:**

BUN level, CRP level, BNP level, average RU volume, and UCR are independent risk factors for PDAP treatment failure. The clinical risk score system based on these five independent risk factors can accurately predict the risk of treatment failure in PDAP individuals.

## Introduction

Peritoneal dialysis (PD) is an economically effective treatment for end-stage renal disease (ESRD) individuals, with advantages such as delaying the decline of residual renal function, maintaining hemodynamic stability, effectively removing middle molecule toxins, and providing individuals with a more independent and freer lifestyle [1, 2]. In the past decade, the number of PD individuals in China has sharply increased, making China the country with the highest number of PD individuals [3]. PD requires long-term placement of a subcutaneous tunnel, and due to individuals’ lack of aseptic consciousness, it can easily cause peritoneal dialysis-associated peritonitis (PDAP). PDAP is a common and serious complication of PD, and it is an important reason for treatment failure that can increase individuals’ risk of death [4, 5]. Studies have shown that a poor prognosis of PDAP can lead to treatment failure in 20% of PD individuals; and among individuals who die after receiving PD, 6% of them die from PDAP [5]. A 10-year single-center study in Taiwan showed that the incidence rate of PDAP was 0.25 times/patient·year (1 time/47.69 months), and the refractory rate was 14.2% (27/190) [6]. Another study reported that for every increase of 0.5 times/(patient/year) in PDAP, the risk of death can increase by 4–11% [7, 8]. Therefore, analyzing PDAP prognostic factors is of great significance for the early clinical identification and implementation of preventive measures.

A risk scoring system is a quantitative risk assessment tool that is widely used in disease diagnosis and patient prognosis research. It can help medical staff systematically screen high-risk individuals based on different risk levels to take targeted preventive measures [9]. Various risk factors may affect the treatment failure of PDAP, and different studies have reported different risk factors [10–12]. For example, some studies have reported that diabetes [13], serum albumin [14], and other factors have predictive value for PDAP prognosis, while other studies believe that these factors cannot be used as outcome predictors for PDAP [15, 16]. Therefore, further research is needed to identify potential predictors of PDAP. Based on the summary of previous research conclusions and logistic regression analysis, this study aimed to identify the influencing factors of PDAP treatment outcome and construct a clinical risk scoring system for PDAP treatment failure, which is expected to predict the adverse outcomes of PDAP individuals and provide guidance for early intervention for such individuals.

## Study objectives and methods

### Study subjects

Employed a strategy of recruiting patients who met specific criteria to select the case and control groups, a retrospective analysis was conducted on 161 PDAP prevalent individuals admitted to our hospital. One patient was excluded due to unclear records of peritonitis occurrences, and three were excluded due to missing contamination information. Ultimately, 157 individuals were included for statistical analysis.

 Inclusion criteria: Individuals diagnosed with PDAP meeting any two of the following three criteria in the International Society for Peritoneal Dialysis Guidelines [8]: (1) Clinical features consistent with peritonitis, including abdominal pain or cloudy dialysis fluid; (2) White blood cell count > 100/µL or > 0.1 × 10^9^/L (after at least 2 h of dwell time) in the dialysis effluent, with polymorphonuclear cells accounting for > 50%;(3) Growth of pathogenic microorganisms in the dialysis fluid culture. In addition, individuals aged ≥ 18 years and with at least one episode of PDAP were included. The markers listed were all collected during the peritonitis episode. Exclusion criteria: Individuals with concurrent tuberculosis or other chronic infectious diseases, and those with incomplete clinical data. All individuals or their legal guardians signed informed consent forms to voluntarily participate in this study.

### Study groups

**Fig. 1:**
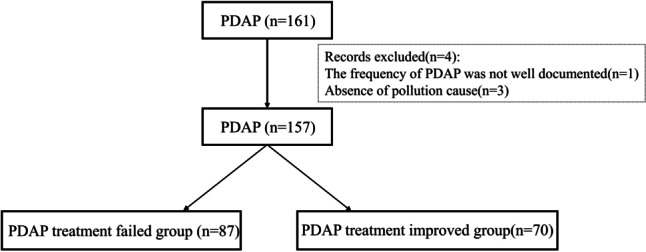
Flowchart of the cohort study

According to the different clinical outcomes of PDAP, individuals were divided into a treatment-improved group (n = 70) and a treatment failure group (n = 87), as shown in the flowchart in Fig. 1.

The main outcome of interest in this study was treatment failure of PDAP, defined as removal or repositioning (may increase the risk of complications and infections) of the catheter, or death after treatment. The criteria for treatment improved were as follows: patients were followed up at least six months after completing the full course of antibiotic treatment. Clinical symptoms improved, white blood cell count in the dialysis effluent < 1 × 10^8^/L, and negative dialysis effluent culture.

### Data collection

A retrospective survey was conducted to collect patient data, including age, gender, history of underlying diseases (diabetes), duration of peritoneal dialysis, contamination reasons (operational errors: improper equipment cleaning or failure to maintain sterile conditions during handling), results of peritoneal dialysis fluid microbiological culture, number of peritonitis occurrences, and laboratory test results (hemoglobin, albumin, blood urea nitrogen (BUN), C-reactive protein (CRP), blood phosphorus, blood uric acid, blood white blood cell count, blood lymphocyte count, blood neutrophil count, platelet count, procalcitonin, B-type natriuretic peptide (BNP), blood parathyroid hormone, left ventricular ejection fraction, peritoneal dialysis effluent white blood cell count, residual renal function eGFR, average residual urine (RU) volume, urea clearance rate (UCR) KT/V), these laboratory test results were all measured at the onset of peritonitis. Patient outcomes after PDAP treatment were followed up and recorded.

### Statistical analysis

SPSS 26.0 was used to analyze the data. Quantitative data that conformed to normal distribution were expressed as mean ± standard deviation, and between-group comparisons were made utilizing unpaired t-test. Quantitative data that did not conform to normal distribution were expressed as median (interquartile range), and between-group comparisons were made utilizing the Wilcoxon Mann-Whitney test. Categorical data were expressed as percentages and compared utilizing the chi-square test. Variables with *P* ≤ 0.05 (two-tailed) were considered statistically significant, while variables with *P* > 0.05 were excluded. Continuous variables were analyzed utilizing the receiver operating characteristic (ROC) curve to determine the cutoff value based on Youden’s index (sensitivity + specificity − 1), and converted into binary data. Utilizing treatment failure in PDAP individuals as the dependent variable, univariate logistic regression was used to screen independent variables, and stepwise regression analysis was used for variable selection. Multivariate logistic regression analysis was used to determine the independent risk factors for treatment failure in PDAP individuals. The corresponding scores were assigned based on the regression coefficients of each risk factor, and the ratio of the independent risk factor B value to the minimum B value was calculated to determine the score. If the ratio was < 1.5, the score was assigned as 1, and if the ratio was ≥ 1.5, the score was assigned as 2, to determine the score of each factor. The sum of the scores of each risk factor was the total risk score of the patient. The diagnostic efficiency of the scoring system was evaluated by the area under the ROC curve (AUC).

## Results

### Comparison of general data of patients

The results of the statistical analysis of the general clinical data of the 157 patients included in this study are shown in Table 1. Except for contamination, peritoneal fluid culture results, BUN level, CRP level, BNP level, average RU volume, and UCR, which showed significant differences (*P* < 0.05), there were no significant differences in other variables (*P* > 0.05).

**Table 1:** Comparison of general data between the two groups

	Treatment improved group (*n* = 70)	Treatment failed group (*n* = 87)	t/df value	*P* value
**Age (year)**	54.79 ± 13.00	57.91 ± 11.70	-1.582	0.116
**Gender**				
** Male**	35 (50.0%)	39 (44.8%)	1.000	0.519
** Female**	35 (50.0%)	48 (55.2%)		
**Diabetes**			1.000	0.915
** Yes**	14 (20.0%)	18 (20.7%)		
** No**	56 (80.0%)	69 (79.3%)		
**Duration of peritoneal dialysis (M)**	46.44 ± 35.08	55.20 ± 31.82	-1.637	0.104
**Contamination**			3.000	**0.036**
** Unknown**	47 (67.1%)	49 (56.3%)		
** Operational errors**	4 (5.7%)	16 (18.4%)		
** Diarrhea**	8 (11.4%)	15 (17.2%)		
** Other**	11 (15.7%)	7 (8.0%)		
**Peritoneal fluid culture results**			4.000	**< 0.001**
** Negative**	29 (41.4%)	26 (29.9%)		
** Gram-negative bacteria**	4 (5.7%)	10 (11.5%)		
** Gram-positive bacteria**	36 (51.4%)	12 (13.8%)		
** Fungus**	0 (0.0%)	39 (44.8%)		
** Other**	1 (1.4%)	0 (0.0%)		
** Number of peritonitis occurrences**	2.11 ± 1.44	2.41 ± 1.54	-1.248	0.214
** Hemoglobin (g/L)**	95.27 ± 21.54	93.95 ± 21.33	0.383	0.702
** Serum creatinine (umol/L)**	825.69 ± 272.00	819.38 ± 259.29	0.148	0.882
** BUN (mmol/L)**	17.59 ± 7.39	14.82 ± 6.54	2.490	**0.014**
** CRP (mg/L)**	86.12 ± 63.23	131.64 ± 90.18	-3.574	**< 0.001**
** Serum phosphate (mmol/L)**	1.75 ± 1.16	1.66 ± 0.57	0.582	0.561
** BUA (umol/L)**	356.79 ± 92.55	368.99 ± 96.39	-0.802	0.424
** WBC (×10^9/L)**	10.81 ± 4.81	11.96 ± 5.40	-1.393	0.165
** Blood lymphocyte count (×10^9/L)**	1.12 ± 1.29	0.84 ± 0.42	1.754	0.083
** Blood neutrophil count (×10^9/L)**	8.99 ± 4.92	10.32 ± 5.09	-1.643	0.102
** Platelet count (×10^9/L)**	266.37 ± 112.19	288.31 ± 141.13	-1.059	0.291
** Procalcitonin (ng/mL)**	19.04 ± 51.15	12.48 ± 27.11	0.969	0.335
** BNP (pg/mL)**	1069.50 (203.80, 2797.25)	264.80 (64.50, 1304.00)	3.363	**0.001**
** PTH (µg/L)**	455.36 ± 434.84	508.49 ± 429.53	-0.766	0.445
** EF (%)**	61.54 ± 9.60	58.47 ± 9.82	1.968	0.051
** White blood cell count in peritoneal dialysate (×10^6/L)**	2924.96 ± 4480.11	2602.52 ± 4166.78	0.466	0.642
** Residual renal function eGFR (mL/min)**	6.44 ± 2.94	6.21 ± 3.19	0.464	0.644
** Average RU volume (mL)**	446.14 ± 135.31	271.16 ± 161.50	7.246	**< 0.001**
** UCR KT/V (/week)**	2.09 ± 0.21	1.83 ± 0.20	7.847	**< 0.001**

### Logistic regression analysis of factors influencing treatment failure in PDAP individuals

 Continuous variables with significant differences (*P* < 0.05) in Comparison of general data of patients section, including BUN level, CRP level, BNP level, average RU volume, and UCR, were included in the analysis. The optimal cutoff values for diagnosis were determined utilizing the Youden’s index based on ROC curve analysis (BUN: 15.3550 mmol/L, CRP: 103.925 mg/L, BNP: 589.400 pg/mL, average RU volume: 375.00 mL, UCR: 1.950), and binary data transformation was completed (Table 2).

**Table 2:** Optimal cutoff values and transformations for continuous variables

Variable	Optimal Cutoff values	Binary conversion
**BUN**	15.3550 mmol/L	If ≤ 15.3550 mmol/L, then n = 1; If > 15.3550 mmol/L, then n = 0
**CRP**	103.925 mg/L	If > 103.925 mg/L, then n = 1; If ≤ 103.925 mg/L, then n = 0
**BNP**	589.400 pg/mL	If ≤ 589.400 pg/mL, then n = 1; If > 589.400 pg/mL, then n = 0
**average RU volume**	375.00 mL	If ≤ 375.00 mL, then n = 1; >375.00 mL, then n = 0
**UCR**	1.950	If ≤ 1.950, then n = 1; If > 1.950, then n = 0

Seven variables, including contamination reasons, peritoneal fluid culture results, BUN level, CRP level, BNP level, average RU volume, and UCR, were included in the univariate logistic regression analysis (Table 3).

**Table 3:** Univariate logistic regression analysis of treatment failure for PDAP

Variable	B value	S.E	Wald	df	*P*	Exp(B)
**Contamination**						
** Unknown**			7.873	3	**0.049**	
** Operational errors**	1.345	0.595	5.105	1	**0.024**	3.837
** Diarrhea**	0.587	0.483	1.476	1	0.224	1.798
** Other**	-0.494	0.525	0.885	1	0.347	0.610
**Peritoneal fluid culture results**						
** Negative**			10.419	4	**0.034**	
** Gram-negative bacteria**	1.025	0.650	2.486	1	0.115	2.788
** Gram-positive bacteria**	-0.989	0.429	5.319	1	**0.021**	0.372
** Fungus**	21.312	6436.026	0.000	1	0.997	1801875786.257
** Other**	-21.094	40192.970	0.000	1	1.000	0.000
** BUN ≤ 15.3550 mmol/L**	0.988	0.331	8.897	1	**0.003**	2.686
** CRP > 103.925 mg/L**	1.179	0.335	12.377	1	**< 0.001**	3.252
** BNP ≤ 589.400 pg/mL**	1.318	0.339	15.089	1	**< 0.001**	3.736
** Average RU volume ≤ 375.00 mL**	2.130	0.371	32.906	1	**< 0.001**	8.414
** UCR ≤ 1.950**	2.191	0.372	34.768	1	**< 0.001**	8.944

Multivariate logistic regression analysis was conducted with PDAP treatment failure as the dependent variable and including contamination reasons, peritoneal fluid culture results, BUN level, CRP level, BNP level, average RU volume, and UCR as independent variables. The variable selection was conducted utilizing stepwise regression analysis, and the independent risk factors for PDAP treatment failure were determined as BUN ≤ 15.3550 mmol/L (Exp(B) = 6.222, *P* = 0.001), CRP > 103.925 mg/L (Exp(B) = 6.675, *P* < 0.001), BNP ≤ 589.400 pg/mL (Exp(B) = 5.411, *P* = 0.001), average RU volume ≤ 375.00 mL (Exp(B) = 5.527, *P* = 0.002), and UCR ≤ 1.950 (Exp(B) = 9.162, *P* < 0.001) (Table 4).

**Table 4:** Multivariate logistic regression analysis of treatment failure for PDAP

variable	B value	S.E	Wald	df	*P*	Exp(B)
**BUN ≤ 15.3550 mmol/L**	1.828	0.536	11.613	1	**0.001**	6.222
**CRP > 103.925 mg/L**	1.898	0.516	13.556	1	**< 0.001**	6.675
**BNP ≤ 589.400 pg/mL**	1.688	0.496	11.603	1	**0.001**	5.411
**Average RU volume ≤ 375.00 mL**	1.710	0.546	9.819	1	**0.002**	5.527
**UCR ≤ 1.950**	2.215	0.588	14.210	1	**< 0.001**	9.162

### Establishment of a clinical risk scoring system for PDAP treatment failure

The independent risk factors were assigned scores based on the regression coefficient (B) obtained from the logistic regression analysis, and the score for each variable was determined based on the ratio of its B value to the minimum B value. Finally, a clinical risk scoring system for PDAP treatment failure was successfully constructed, as shown in Table 5: BUN ≤ 15.3550 mmol/L (1 point), CRP > 103.925 mg/L (1 point), BNP ≤ 589.400 pg/mL (1 point), average RU volume ≤ 375.00 mL (1 point), and UCR ≤ 1.950 (1 point), with a total score of 5 points.

**Table 5:** Clinical risk system composition and its score for treatment failure of PDAP

Variable	B value	Ratio	Score
**BUN ≤ 15.3550 mmol/L**	1.828	1.083	1
**CRP > 103.925 mg/L**	1.898	1.124	1
**BNP ≤ 589.400 pg/mL**	1.688	1.000	1
**Average RU volume ≤ 375.00 mL**	1.710	1.013	1
**UCR ≤ 1.950**	2.215	1.312	1
**Total score**			5

### Validation of the PDAP treatment failure clinical risk scoring system

**Fig. 2:**
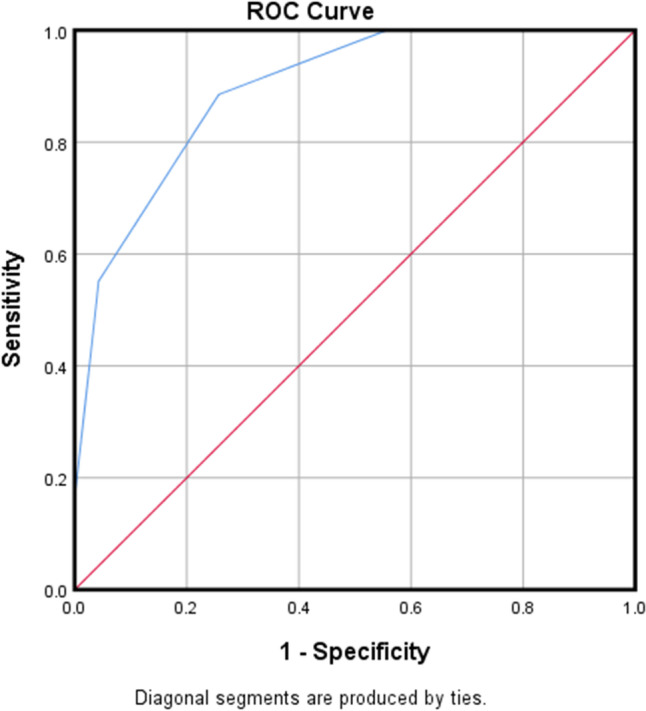
ROC curve of the clinical risk scoring system for PDAP treatment failure

All patients were risk-scored utilizing the aforementioned scoring system, and the diagnostic performance of the risk scoring system was validated through ROC curve analysis. The results are shown in Fig. 2: the AUC of the PDAP treatment failure clinical risk scoring system was 0.895 (95% confidence interval: 0.847–0.943). At the optimal cutoff score of 2.5 points, the sensitivity and specificity were 88.5% and 74.3%, respectively.

## Discussion

With the advancement of PD technology and related equipment, the incidence of PDAP has shown a decreasing trend, and the survival rate of PD individuals has improved. However, PDAP remains a major cause of treatment failure in individuals [17]. Therefore, identifying the risk factors for adverse outcomes of PDAP and avoiding them to reduce its incidence is of great clinical significance. As a form of predictive tool, a scoring system can transform complex clinical and pathological indicators into simple scores to facilitate the prediction of the risk of PDAP treatment failure. Our study results showed that BUN level, CRP level, BNP level, average RU volume, and UCR were independent risk factors for PDAP treatment failure. Furthermore, a clinical risk scoring system was constructed based on the above five factors, which was found to be able to predict the risk of PDAP treatment failure with reasonable accuracy and effectiveness.

Early identification of risk factors for treatment failure in PDAP individuals is a recognized focus in the nephrology community. Previous literature has explored the risk factors for treatment failure in PDAP individuals from various aspects, such as gender, age, intestinal dysfunction, malnutrition, diabetes, and body mass index, but with inconsistent conclusions [18–20]. Studies reported by Chen and others [10] have shown that diabetes is a risk factor for PDAP, and that PDAP is correlated with gender, with male individuals, lower serum albumin levels, gram-negative bacteria, and multiple microbial infections being at higher risk of treatment failure. Additionally, lower UCR is a risk factor for death in PD individuals. However, the general data comparison results of the two groups of individuals in this study showed no significant differences in the basic factors of diabetes and gender among individuals with different outcomes in both groups. Furthermore, the data results in this study showed that the level of gram-positive bacteria in the peritoneal fluid culture results of the group with improved treatment was significantly higher than that in the group with treatment failure (*P* < 0.05). Although there was no significant difference in the gram-negative bacteria results, the level in the treatment failure group was higher than that in the treatment-improved group (*P* = 0.115), which is similar to the results reported by Chen’s study. URC is an indicator for evaluating the peritoneal function of patients [10], and BUN and average RU volume are evaluation indicators for renal function. In this study, the BUN, RU volume, and UCR in the treatment failure group were all significantly lower than those in the treatment-improved group (*P* < 0.05), consistent with the results reported in the aforementioned study [10], and the results of the multivariate logistic regression risk analysis also confirmed this view. In addition, the results of this study showed that factors such as operational errors may also affect the treatment results of PDAP individuals (*P* < 0.05), indicating that clinical practitioners should pay attention to standardized, aseptic operation awareness and avoid medical operational errors.

PDAP is associated with the patient’s systemic inflammation and local inflammation in the abdomen. Individuals with ESRD have serious immune deficiencies and accumulate a large amount of uremic toxins, which stimulate the body to produce inflammatory factors such as TNF-α, IL-6, and IL-1β, leading to sustained microinflammation reaction [21–24]. CRP is an acute-phase reactant protein that is highly sensitive to bacterial infections. When the body is infected or damaged, inflammatory cells will stimulate the liver to synthesize CRP. However, after the body function recovers and the inflammation subsides, the CRP level gradually returns to normal levels [25, 26]. CRP is one of the most sensitive indicators of inflammatory response for diagnosing infectious diseases, and its elevated levels suggest that the body is in an inflammatory or oxidative stress state [27, 28]. Su et al.’s study [29]evaluated the effect of CRP on PDAP individuals and found that if the CRP level of a patient increases during the first year of PD treatment, the patient’s risk of developing PDAP increases after one year. Furthermore, 30–50% of PD individuals may experience elevated CRP levels [30, 31]. A cross-sectional study also confirmed that the baseline CRP level of PD individuals is an independent risk factor for PD treatment failure [31]. In this study, the CRP level in the treatment failure group was significantly higher than that in the treatment-improved group (*P* < 0.05). Similar to the previously reported results, high CRP levels are a risk factor for PDAP treatment failure. BNP level is a kind of peptide neurohormone with natriuretic and diuretic effects. It can inhibit the renin-angiotensin-aldosterone system and sympathetic-adrenal system activity, reduce myocardial cell fibrosis, promote vascular smooth muscle relaxation, dilate blood vessels, and is often used as an indicator of changes in heart function to assist in the diagnosis of heart failure [32–35]. Currently, there is limited research on the relationship between BNP levels and PDAP. The analysis results of general patient information in this study showed that the BNP levels in the treatment improvement group were significantly higher than those in the treatment failure group (*P* < 0.05). At the same time, the multivariate logistic regression risk results showed that BNP levels were an independent risk factor for PDAP treatment failure (*P* < 0.05), indicating that changes in the cardiovascular function of PDAP individuals may lead to treatment failure.

In this study, a clinical risk scoring system for predicting the failure of PDAP treatment was preliminarily constructed by collecting clinical data, and the effectiveness of the risk scoring system in predicting the failure of PDAP treatment was validated. The area under the ROC curve was 0.895, indicating that the risk prediction scoring system has good predictive diagnostic performance. This system can help clinical workers identify high-risk groups for PDAP treatment failure as early as possible, providing a theoretical basis for timely, rational, and effective treatment and care measures for such groups. In addition, the relevant factors included in the scoring system designed in this study are easily collected clinical indicators, which are simple, quantifiable, and feasible, and can effectively improve compliance with preventive measures. However, this study also has some limitations: First, it was a retrospective case-control study, and the factors that may affect the study’s inclusion were obtained based on clinical experience and reference to relevant literature, which may result in selection bias due to the lack of strict scoring criteria and the existence of subjective factors. Second, this study was a single-center study with small sample size, and the conclusions need to be verified and consolidated by larger multi-center retrospective or prospective cohort studies. In addition, the history of underlying diseases of patients in this paper only makes statistics on diabetes, without considering other comorbidities. We also did not collect data on the number of patients with peritonitis combined with exit site or tunnel infections. Hence, we will analyze more factors that may affect patients in the future.

## Data Availability

All data found in the study has been included in the study.

## References

[1] Bello AK, Bello AK, Okpechi IG, Osman MA, Cho Y, Cullis B, Htay H, Jha V, Makusidi MA, McCulloch M, Shah N, Wainstein M, Johnson DW (2022). Epidemiology of peritoneal dialysis outcomes. Nat Rev Nephrol.

[2] Auguste BL, Bargman JM (2023). Peritoneal Dialysis prescription and adequacy in clinical practice: Core Curriculum 2023. Am J Kidney Dis.

[3] Yu X, Yang X (2015). Peritoneal dialysis in China: meeting the challenge of chronic kidney failure. Am J Kidney Dis.

[4] Ye H, Ye H, Zhou Q, Fan Li, Guo Q, Mao H, Huang F, Yu X, Yang X (2017). The impact of peritoneal dialysis-related peritonitis on mortality in peritoneal dialysis patients. BMC Nephrol.

[5] Boudville N, Boudville N, Kemp A, Clayton P, Lim W, Badve SV, Hawley CM, McDonald SP, Wiggins KJ, Bannister KM, Brown FG, Johnson DW (2012). Recent peritonitis associates with mortality among patients treated with peritoneal dialysis. J Am Soc Nephrol.

[6] Wang HH, Wang H-H, Huang C-H, Kuo M-C, Lin S-Y, Hsu C-H, Lee C-Y, Chiu Y-W, Chen Y-H, Lu P-L (2019). Microbiology of peritoneal dialysis-related infection and factors of refractory peritoneal dialysis related peritonitis: a ten-year single-center study in Taiwan. J Microbiol Immunol Infect.

[7] Li PK (2016). ISPD PeritonitisRecommendations Update on Prevention and Treatment. Perit Dial Int.

[8] Li PK (2022). ISPD peritonitis guideline recommendations: 2022 update on prevention and treatment. Perit Dial Int.

[9] Yang HI, Yang H-I, Yuen M-F, Chan H-Y, Han K-H, Chen P-J, Kim D-Y, Ahn S-H, Chen C-J, Wong V-S, Seto W-K (2011). Risk estimation for hepatocellular carcinoma in chronic hepatitis B (REACH-B): development and validation of a predictive score. Lancet Oncol.

[10] Chen HL, Tarng DC, Huang LH (2019). Risk factors associated with outcomes of peritoneal dialysis in Taiwan: an analysis using a competing risk model. Med (Baltim).

[11] Htay H, Htay H, Cho Y, Pascoe EM, Hawley C, Clayton PA, Borlace M, Badve SV, Sud K, Boudville N, Chen JHC, Sypek M, Johnson DW (2020). Multicentre registry data analysis comparing outcomes of culture-negative peritonitis and different subtypes of culture-positive peritonitis in peritoneal dialysis patients. Perit Dial Int.

[12] Htay H, Htay H, Cho Y, Pascoe EM, Darssan D, Nadeau-Fredette A-C, Hawley C, Clayton PA, Borlace M, Badve SV, Sud K, Boudville N, McDonald SP, Johnson DW (2018). Center Effects and Peritoneal Dialysis Peritonitis Outcomes: analysis of a National Registry. Am J Kidney Dis.

[13] Nochaiwong S, Nochaiwong S, Ruengorn C, Koyratkoson K, Thavorn K, Awiphan R, Chaisai C, Phatthanasobhon S, Noppakun K, Suteeka Y, Panyathong S, Dandecha P, Chongruksut W, Nanta S, Ruanta Y, Tantraworasin A, Wongsawat U, Praseartkul B, Sattaya K, Busapavanich S (2018). A clinical risk Prediction Tool for Peritonitis-Associated Treatment failure in peritoneal Dialysis patients. Sci Rep.

[14] Meng L (2022). [Development and validation of a prediction model for treatment failure in peritoneal dialysis-associated peritonitis patients: a multicenter study]. Nan Fang Yi Ke Da Xue Xue Bao.

[15] Meng LF, Meng L-F, Yang L-M, Zhu X-Y, Zhang X-X, Li X-Y, Zhao J, Liu S-C, Zhuang X-H, Luo P, Cui W-P (2020). Comparison of clinical features and outcomes in peritoneal dialysis-associated peritonitis patients with and without diabetes: a multicenter retrospective cohort study. World J Diabetes.

[16] Liu X (2021). Novel predictors and risk score of treatment failure in peritoneal Dialysis-related Peritonitis. Front Med (Lausanne).

[17] Campbell DJ, Campbell DJ, Craig JC, Mudge DW, Brown FG, Wong G, Tong A (2016). Patients’ perspectives on the Prevention and Treatment of Peritonitis in Peritoneal Dialysis: a semi-structured interview study. Perit Dial Int.

[18] Tsai CC, Tsai C-C, Lee J-J, Liu T-P, Ko W-C, Wu C-J, Pan C-F, Cheng S-P (2013). Effects of age and diabetes mellitus on clinical outcomes in patients with peritoneal dialysis-related peritonitis. Surg Infect (Larchmt).

[19] Wu HH, Wu H-H, Li I-J, Weng C-H, Lee C-C, Chen Y-C, Chang M-Y, Fang J-T, Hung C-C, Yang C-W, Tian Y-C (2013). Prophylactic antibiotics for endoscopy-associated peritonitis in peritoneal dialysis patients. PLoS ONE.

[20] Hsieh YP, Chang CC, Wen YK, Chiu PF, Yang Y (2014). Predictors of peritonitis and the impact of peritonitis on clinical outcomes of continuous ambulatory peritoneal dialysis patients in Taiwan–10 years’ experience in a single center. Perit Dial Int.

[21] Pashenkov MV, Murugina NE, Budikhina AS, Pinegin BV (2019). Synergistic interactions between NOD receptors and TLRs: mechanisms and clinical implications. J Leukoc Biol.

[22] Stenvinkel P (2005). IL-10, IL-6, and TNF-alpha: central factors in the altered cytokine network of uremia–the good, the bad, and the ugly. Kidney Int.

[23] Sun T, Sun T, Sakata F, Ishii T, Tawada M, Suzuki Y, Kinashi H, Katsuno T, Takei Y, Maruyama S, Mizuno M, Ito Y (2019). Excessive salt intake increases peritoneal solute transport rate via local tonicity-responsive enhancer binding protein in subtotal nephrectomized mice. Nephrol Dial Transplant.

[24] Memoli B, Memoli B, Romano G, D’Arcangelo R, Del Prete M, Esposito P, Procino A, Cuomo V, Bisesti V, Capuano A, Andreucci VE (2004). The role of interleukin-6 and of its soluble receptors in the biocompatibility of dialysis treatment. Semin Nephrol.

[25] Pathak A, Agrawal A (2019). Evolution of C-Reactive protein. Front Immunol.

[26] Windgassen EB, Funtowicz L, Lunsford TN, Harris LA, Mulvagh S (2011). L. C-reactive protein and high-sensitivity C-reactive protein: an update for clinicians. Postgrad Med.

[27] Metwally K, Fouad T, Assem M, Abdelsameea E, Yousery M (2018). Predictors of spontaneous bacterial peritonitis in patients with cirrhotic ascites. J Clin Transl Hepatol.

[28] Osimo EF, Baxter LJ, Lewis G, Jones PB, Khandaker GM (2019). Prevalence of low-grade inflammation in depression: a systematic review and meta-analysis of CRP levels. Psychol Med.

[29] Su YJ, Liao SC, Cheng BC, Hwang JC, Chen JB (2013). Increasing high-sensitive C-reactive protein level predicts peritonitis risk in chronic peritoneal dialysis patients. BMC Nephrol.

[30] Herzig KA, HERZIG KARENANN, PURDIE DAVIDMICHAEL, CHANG WENDY, BROWN ALLISONMARGARET, HAWLEY CARMELMARY, CAMPBELL SCOTTBRYAN, STURTEVANT JOANNAMARY, ISBEL NICOLEMAREE, NICOL DAVIDLAWRENCE, JOHNSON DAVIDWAYNE (2001). Is C-reactive protein a useful predictor of outcome in peritoneal dialysis patients?. J Am Soc Nephrol.

[31] Liu SH, Liu S-H, Li Y-J, Wu H-H, Lee C-C, Lin C-Y, Weng C-H, Chen Y-C, Chang M-Y, Hsu H-H, Fang J-T, Hung C-C, Yang C-W, Tian Y-C (2014). High-sensitivity C-reactive protein predicts mortality and technique failure in peritoneal dialysis patients. PLoS ONE.

[32] Ning C, Zheng Y, Li J, Liu M, Fang Z (2020). Effects of recombinant human brain natriuretic peptide in patients with acute myocardial infarction undergoing percutaneous coronary intervention: a systematic review and meta-analysis. Med (Baltim).

[33] Chen S, Chen S, Redfors B, O’Neill BP, Clavel M-A, Pibarot P, Elmariah S, Nazif T, Crowley A, Ben-Yehuda O, Finn MT, Alu MC, Vahl TP, Kodali S, Leon MB, Lindman BR (2020). Low and elevated B-type natriuretic peptide levels are associated with increased mortality in patients with preserved ejection fraction undergoing transcatheter aortic valve replacement: an analysis of the PARTNER II trial and registry. Eur Heart J.

[34] Touzot M (2020). Mathematical model to predict B-type natriuretic peptide levels in haemodialysis patients. Nephrol (Carlton).

[35] Kadri AN, Kadri AN, Kaw R, Al-Khadra Y, Abumasha H, Ravakhah K, Hernandez AV, Tang WHW (2018). The role of B-type natriuretic peptide in diagnosing acute decompensated heart failure in chronic kidney disease patients. Arch Med Sci.

